# Bicuspid Aortic Valve and Dilatation of the Ascending Aorta

**DOI:** 10.1155/2012/142697

**Published:** 2012-11-21

**Authors:** Martin Misfeld, Ani C. Anyanwu, Adrian H. Chester

**Affiliations:** ^1^Department of Cardiac Surgery, Leipzig Heart Center, University of Leipzig, 04289 Leipzig, Germany; ^2^Department of Cardiothoracic Surgery, Mount Sinai Hospital, New York, NY 10029, USA; ^3^Heart Science Centre, Harefield Hospital, Harefield UB9 6JH, UK

The aortic valve is a complex structure that relies on coordinated interactions between its component parts. It is now established that the cells within, and on the cusps, play a key role in the optimal performance of the valve within the unique mechanical environment in which it is required to function. In the majority of individuals, the valve opens and closes over 3 billion times without any significant problems. The importance of the relationship between structure and function is illustrated by the inability of a valve that has structural malformations to function normally throughout life. Valves that have only two valve functional cusps, as opposed to the usual three, are associated with accelerated disease and failure at a young age. 

The presence of bicuspid aortic valves represents a significant issue, since they are estimated to occur in 1-2% of live births. In addition to the development of stenotic disease at a young age, the presence of a bicuspid valve is also associated with diseases of the aorta. This special issue contains a number of review articles and original manuscripts that specifically address the issues related with bicuspid aortic valves and associated dilatation of the ascending aorta. 

The papers include two general reviews of the subject that covers embryology, structure genetics of bicuspid valve formation, clinical aspects of bicuspid valves, and highlights of where gaps in our knowledge exist. Subsequent contributions focus more specifically on the morphology of bicuspid valves and how different valve configurations relate to different pathophysiological outcomes with regard to the defects in the medial wall of the ascending aorta, dilation of the proximal aorta, and their effect on the flow patterns across the valve. The association between the presence a bicuspid aortic valve and thoracic aortic aneurysms is the subject of another paper. Whiles these are two separate entities, they share common developmental pathways. The paper sets out to explore how these shared genetic pathways can manifest individual patient populations. The management of the patients with a bicuspid aortic valve, aortic aneurysms, or both, represents a specific clinical challenge. These challenges are assessed with regard to the anatomical differences in the phenotype of the bicuspid valve, the pathophysiology, and natural history of the patients. 

In addition to the review articles on the bicuspid valve and aortic aneurysms, this issue also contains papers that present original data. S. A. Mohamed et al. examine the expression of nitric oxide synthase in aortic aneurysms from patients with bicuspid and tricuspid valves. The regional differences reported on nitric oxide synthase expression in the two groups suggest dysregulation of the nitric oxide system in patients with bicuspid aortic valves. This study is of interest due to the reported incidence of bicuspid valve formation in mice lacking the endothelial nitric oxide synthase gene. The second original study by J. Benedik et al. examines parameters relating to thickness of the aortic wall, diameters at different regions of the aortic root wall, and the cohesion of the aortic wall of 190 patients undergoing valve surgery. While some differences existed in the geometry of the aortic root and wall thickness, they report no difference in the tensile strength curves between bicuspid and tricuspid valves, suggesting that the pathology of the aorta and valve is unrelated. 

The only current treatment for individuals who develop disease in their bicuspid valve or aortic aneurysms remains surgical repair or replacement of the valve and or aorta. The current guidelines for treatment, and importantly when to treat asymptomatic patients, are reviewed by C. D. Etz et al. in the last paper of this issue. 

The papers in this issue are a reflection of the different areas of research and treatment that are pertinent to understanding the development and functional consequences of having a bicuspid aortic valve. Additional studies are required to understand the genetics of this condition and why bicuspid valves have an association with aortic aneurysms. Ultimately, this will lead to more effective treatment of patients affected with these conditions. 

*Martin Misfeld* *Ani C. Anyanwu* *Adrian H. Chester*

